# Overview of Social Cognitive Dysfunctions in Rare Developmental Syndromes With Psychiatric Phenotype

**DOI:** 10.3389/fped.2018.00102

**Published:** 2018-05-03

**Authors:** Aurore Morel, Elodie Peyroux, Arnaud Leleu, Emilie Favre, Nicolas Franck, Caroline Demily

**Affiliations:** ^1^Scientific Brain Training, Reference Center for Rare Diseases GénoPsy, CH Le Vinatier, UMR 5229, Université Lyon 1, CNRS, Lyon, France; ^2^Reference Center for Rare Diseases GénoPsy, SUR/CL3R: Service Universitaire de Réhabilitation, CH Le Vinatier, UMR 5229, Université Lyon 1, CNRS, Lyon, France; ^3^Centre des Sciences du Goût et de l’Alimentation, AgroSup Dijon, INRA, Université Bourgogne Franche-Comté, CNRS, Dijon, France; ^4^Reference Center for Rare Diseases GénoPsy, CH Le Vinatier, UMR 5229, Université Lyon 1, CNRS, Lyon, France; ^5^Centre ressource de réhabilitation psychosociale et de remédiation cognitive, CH Le Vinatier, Lyon et UMR 5229 (CNRS and Université Lyon), Lyon, France

**Keywords:** social cognition, facial emotion recognition, theory of mind, systematic review, genetics, neurodevelopmental disorders

## Abstract

Rare neurodevelopmental syndromes often present social cognitive deficits that may underlie difficulties in social interactions and increase the risk of psychosis or autism spectrum disorders. However, little is known regarding the specificities of social cognitive impairment across syndromes while it remains a major challenge for the care. Our review provides an overview of social cognitive dysfunctions in rare diseases associated with psychiatric symptoms (with a prevalence estimated between 1 in 1,200 and 1 in 25,000 live births: 22q11.2 deletion syndrome, Angelman syndrome, Fragile X syndrome, Klinefelter syndrome, Prader–Willi syndrome, Rett syndrome, Smith–Magenis syndrome, Turner syndrome, and Williams syndrome) and shed some light on the specific mechanisms that may underlie these skills in each clinical presentation. We first detail the different processes included in the generic expression “social cognition” before summarizing the genotype, psychiatric phenotype, and non-social cognitive profile in each syndrome. Then, we offer a systematic review of the social cognitive abilities and the disturbed mechanisms they are likely associated with. We followed the PRISMA process, including the definition of the relevant search terms, the selection of studies based on clear inclusion, and exclusion criteria and the quality appraisal of papers. We finally provide insights that may have considerable influence on the development of adapted therapeutic interventions such as social cognitive training (SCT) therapies specifically designed to target the psychiatric phenotype. The results of this review suggest that social cognition impairments share some similarities across syndromes. We propose that social cognitive impairments are strongly involved in behavioral symptoms regardless of the overall cognitive level measured by intelligence quotient. Better understanding the mechanisms underlying impaired social cognition may lead to adapt therapeutic interventions. The studies targeting social cognition processes offer new thoughts about the development of specific cognitive training programs, as they highlight the importance of connecting neurocognitive and SCT techniques.

## Introduction

Neurodevelopmental syndromes are genetic abnormalities frequently associated with behavioral and/or psychiatric phenotypes. Notably, these syndromes increase the difficulties in social interactions and present a high risk to develop psychosis or autism spectrum disorders (ASDs) (1–3). While social difficulties are generally associated with several comorbidities of genetic conditions, such as intellectual impairments, facial dysmorphology, speech problems, and psychotic symptoms, the high prevalence of social cognitive disorders recently received a growing interest. The aim here is thus to provide an overview of social cognitive abilities in developmental syndromes associated with psychiatric symptoms and shed some light on the specific mechanisms that may underlie these skills in each clinical presentation.

We focus on syndromes with a psychiatric phenotype (psychosis and/or ASD) and a prevalence estimated between 1 in 1,200 and 1 in 25,000 live births: 22q11.2 deletion syndrome (22q11.2DS) (4–6), Angelman syndrome (AS), Fragile X syndrome (FXS) (7), Klinefelter syndrome (KS), Prader–Willi syndrome (PWS) (8, 9), Rett syndrome (RS), Smith–Magenis syndrome (SMS) (10), Turner syndrome (TS), and Williams syndrome (WS) (11). We first detail the different processes included in the generic expression “social cognition” before summarizing the genotype, psychiatric phenotype, and non-social cognitive profile in each syndrome. Then, we offer a systematic review of the social cognitive abilities and the disturbed mechanisms they are likely associated with. We finally provide insights that may have considerable influence on the development of adapted therapeutic interventions such as social cognitive training (SCT) therapies specifically designed to target the psychiatric phenotype.

### Social Cognition

**Figure 1:**
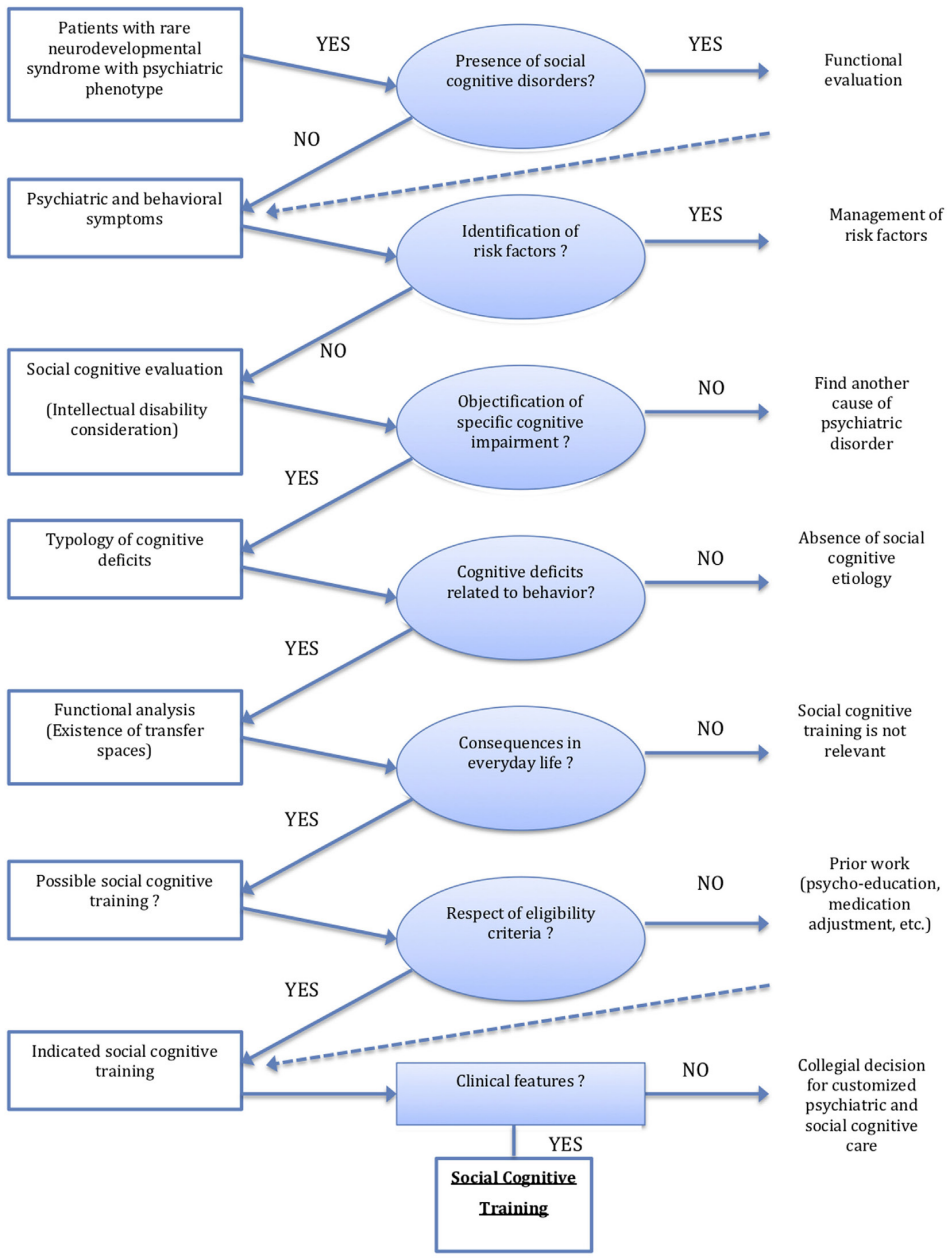
Organizational decision chart of social cognitive training in rare neurodevelopmental syndromes with psychiatric phenotype.

Social cognition is defined as the ability to understand, perceive, and interpret information about other people and ourselves in a social context. This includes abilities such as emotion recognition, theory of mind (ToM), attributional style, and social perception and knowledge (Figure 1). Broadly speaking, social cognition includes a wide range of processes that allow people to rapidly, effortlessly, and flexibly perceive and interpret rapidly changing social information, and respond appropriately to social stimuli. Besides, this ability gives meaning to the actions of others. More specifically, social cognition is an “umbrella concept” that includes many heterogeneous cognitive dimensions, such as emotional information processing, social perception and knowledge, ToM, and attributional bias (12).

#### Emotional Information Processing

Emotional information about others is conveyed by complex signals such as prosody and emotional expressions from the face or the body (13). Regarding emotional expressions, Ekman (14) described six basic emotions (happiness, sadness, fear, disgust, anger, and surprise) that can be easily discriminated from one another depending on different facial patterns (14, 15). Such categorical perception requires the processing of fine face changes and specific observation strategies.

#### Theory of Mind

An individual has a ToM if he imputes mental states to himself and others. A system of inferences of this kind is properly viewed as a theory because such states are not directly observable, and the system can be used to make predictions about the behavior of others (16–18). This theory comprises two dimensions: cognitive ToM (beliefs, intentions, etc.) and affective ToM (emotional state, knowledge of emotion, etc.) (19, 20). The ToM is subtended by first- and second-order representations. The first-order representation is defined as the ability to understand another person’s mental state. The second-order representation is the ability to understand what one person thinks about another person’s thoughts. These levels are both supported by the primary ability to understand how and why an individual does something (e.g., understanding motor action and eye direction) (16).

#### Attributional Style

Attribution refers to the way people explain their own and other people’s behaviors (21), but individuals are not objective perceivers and sometimes attribution does not properly match reality. Some individuals suffer from attribution bias, i.e., they systematically over- and under-use the available information, which leads to a misinterpretation of the world they live in.

#### Social Perception and Knowledge

Social perception refers to the understanding of social roles and rules that typically appear in social situations. Social knowledge is the awareness of the behavior expected in different social contexts and interactions (12). This knowledge consists of social experience, education, and ritualized practices that are not necessarily explicitly communicated. These processes are the basis of an accurate analysis of another person’s intentions, desires, or emotional states; more generally, it is linked to ToM.

### Selected Neurodevelopmental Syndromes

The Table 1 presents a summary of the main medical, psychiatric, and social characteristics of the neurodevelopmental syndromes cited in this review.

**Table 1:** Summary presentation of the main medical and social characteristics of the neurodevelopmental syndromes cited in this review.

Syndrome	Chromosome	Prevalence	Medical characteristics	Main characteristics of social behavior
22q11.2 DS	22q11.2	1 in 2,000–4,000 births (22, 23)	Characteristic facial dysmorphology, mainly congenital heart disease, velopharyngeal insufficiency, cleft palate, neuromuscular problems, hypoparathyroidism, and thymic hypoplasia	Withdrawal [but normal social motivation and adaptive behavior according to Ref. (24)] Difficulties to maintain relationships High risk of development of psychotic disorders (25, 26)
Angelman syndrome	15q11q13	1 in 10,000–12,000 births (27, 28)	Microcephaly, epilepsy, feeding problems	High levels of social approach behavior toward familiar (e.g., their mothers) and unfamiliar adults, strong enthusiasm for adult attention (29, 30) Autistic behaviors such as balance and movement problems, manual stereotypy such as hand-flapping (31), absence of eye contact, fascination for certain objects, and intolerance to change (32) Frequent laughter can be caused by a minimal stimulus and is often inappropriate
Fragile X syndrome	X	1 in 1,500 males and 1 in 2,500 females (33)	Characteristic facial dysmorphology. Macroorchidism, hyperextensible metacarpophalangeal joints, curvature of the spine (scoliosis), seizures (epilepsy), heart murmurs	Withdrawal, anxious Autistic behaviors including avoidance of eye contact (34, 35), sensory hypersensitivity, stereotypical behavior, hand flapping, echolalia, and language delay (36, 37)
Klinefelter syndrome	X aneuploidy	1 in 667 births (38)	Tall stature with disproportionally long legs and arms. Hypogonadism, gynecomastia, infertility. Comorbid symptoms such as taurodontism, osteopenia, breast cancer, thyroid dysfunction, and chronic autoimmune disease	High social anxiety levels and reduced social assertiveness (39). But level of social motivation is not impaired (40) Men with a prenatal diagnostic may present with fewer social difficulties, which may be due to an increased prenatal support to the family (40)
Prader–Willi syndrome	15q11–q13	1 in 15,000 to 20,000 births (41)	Characteristic facial dysmorphy. Neonatal hypotonia and feeding difficulties. Hyperphagia, obsession with food, hypogonadism	Maladaptive behaviors such as outbursts, self-mutilation, impulsivity, ritualistic behaviors, repetitive conversation topics, and difficulties with routine change
Rett syndrome	X	1 in 10,000 newborn girls (42)	Partial or complete loss of hand skills, apraxia, spasticity, scoliosis, abnormal breathing patterns, and seizures	Atypical socio-communicative pattern that develops prior to the period of regression: the use body movements, gestures, eye gazes, vocalization, and production of words depending on the context (expressing discomfort or happiness, making choices, requesting objects, performing activities, focusing attention, and socializing) (43–45)
Smith–Magenis syndrome	17p11.2	1 in 25,000 births (46)	Characteristic facial dysmorphy. Brachycephaly, midface hypoplasia, prognathism, hoarse voice, infantile hypotonia, chronic otitis, sleep disorders	Strong desire for social interaction, good eye contact (47, 48) Maladaptive behaviors such as self-injury (including wrist biting, skin picking and head banging), temper tantrums, body rocking, contrasting with challenging behavior, hyperactivity, insert their hand in their mouth or objects into body orifices, sometimes with public masturbation Stereotypical behaviors including repetitive questions, routine, and the tendency to bring about recurring subjects
Turner syndrome	Complete or partial absence of one X chromosome	1 in 2,000 live female births (49)	Short stature, ovarian failure, sex hormone deficiencies, webbed neck, cardiac malformation, abnormal pubertal development, and amenorrhea with infertility	Shyness, social anxiety, low self-esteem (social acceptance, romantic relationships, etc.), social withdrawal, emotionally less mature than controls (50, 51) [but normal social motivation according to Ref. (52, 53)] Good social knowledge but difficulties to put it into practice in real life, probably due to social anxiety (54)
Williams syndrome	7q11.23	1 in 7,500 births (55)	Characteristic facial features. Cardiovascular disease, hyperopia, strabismus, feeding difficulties, hypercalcemia, joint hypermobility	Difficulties making friends, maladaptive behaviors, non-social anxiety, and difficulties in social reciprocity similar to individuals with Autism spectrum disorder (56) Heterogeneous social skills (57, 58)

## Materials and Methods

This systematic review follows the process developed by the PRISMA statement, including the definition of the relevant search terms, the selection of studies based on clear inclusions and exclusion criteria, and the quality appraisal of papers.

### Search Strategy

A search of the literature was conducted using the electronic database PubMed and Google Scholar, covering the period between first January 1990 and December 2016 and focusing on articles published in English. Broad search terms were used, including “social cognition,” “theory of mind,” “mind reading,” “mentalizing,” “mentalising,” “emotion recognition,” “emotion perception,” “emotion processing,” “affect recognition,” “affect perception,” “affect processing,” “social knowledge,” “attributional style,” “facial emotion,” “auditory emotion,” “gaze processing,” “body posture,” “social cues” in combination with the diagnostic terms “22q11.2 deletion syndrome” (22q11.2DS), “Angelman syndrome,” “Fragile X syndrome” (FXS), “Klinefelter syndrome” (KS), “Prader-Willi syndrome” (PWS), Rett syndrome” (RS), “Smith-Magenis syndrome” (SMS), “Turner syndrome” (TS) and “Williams syndrome” (WS). Reference lists of the retrieved articles were also manually searched for relevant publications. All articles investigating the perception and/or recognition of affective stimuli (facial expression, prosody, and body language), ToM, social knowledge and attributional style in patients with rare neurodevelopmental syndromes were evaluated. Only studies that involved a behavioral task were included. We excluded from our evaluation the studies that only included neuroimaging data, the studies with non-humans and the reviews, and studies that did not directly assessed social cognition such as studies using only a questionnaire assessing social behavior.

A total of 256 articles were initially identified as potential papers for inclusion. After a comprehensive reading by the first author (Aurore Morel), 136 articles were excluded from the analysis (e.g., studies not focused on behavior or duplicates). A total of 120 papers met the inclusion criteria and were eligible for quality appraisal (40 duplicates).

All titles and abstracts of the identified studies were independently assessed by two authors (Elodie Peyroux and Caroline Demily) for inclusion in or exclusion from the systematic review. Papers were excluded when inclusion criteria were not met (disagreements were resolved by discussion).

### Data Analysis

We extracted relevant data from the 132 studies. A meta-analysis of the studies was not feasible given the variations among study designs, social cognitive assessments used, and populations.

A thematic analysis was performed to qualitatively synthesize the data, aiming at identifying specific syndrome patterns. Data were presented in a narrative form. The analytic process was carried on through discussion between the authors (Aurore Morel, Arnaud Leleu, Elodie Peyroux, Emilie Favre, Nicolas Franck, and Caroline Demily).

### Patients

We proposed a review analysis, reporting the number of subjects included, the nature of the control population, the statistical method used, and the results obtained for all current published studies (Tables 2–5).

**Table 2:** Fourteen studies examining social cognition in 22q11.2 deletion syndrome (22q11.2DS).

Reference	*N*	Mean age in years (range)	Full scale intelligence quotient (SD) [range]	Comparison group	Social cognition domain (task)	Data analyses	Main results
(59)	17	17.2	72.5 (12.7)	Typically developing (TD)	Exploring face	ANOVA	Patients with 22q11.2 spent less time observing the eye and mouth region than controls (*p* = 0.009)
(60)	50	11.00 (6–16)	65.80 (9.32) [40–94]	TD	Ability to infer first-order false beliefs (the Smarties task and on the Sally–Ann task) Ability to infer second-order false beliefs Cognitive theory of mind (ToM) (strange stories)	Fisher’s exact test Pearson correlations	Children with 22q11.2 did not show difficulties on the Smarties task (*p* = 0.52) and on the Sally–Ann task (*p* = 0.15) Only younger children with 22q11.2DS showed poorer performance on second-order false-belief tasks and strange stories task (*p* < 0.02)
(61)	24	16.75 (12–21)	75.88 (14.93) [56–115]	TD	Ability to infer second-order false beliefs Affective ToM	ANOVA	Children with 22q11.2DS had difficulties to attribute emotions based on circumstances (*p* = 0.001)
(62)	35	18.2 (9–33)	69.5 (11.3)	TD	Identification of dynamic facial expression (labeling task)	MANCOVA	Individuals with 22q11.2 and controls with typical development and the same age showed similar performance (*p* = 0.81) But participants with 22q11.2 were slower to recognize emotions (*p* < 0.01)
(63)	26	12.36 (8–15)	74.19 (11.99)	“Idiopathic” developmental delay (DD) + TD	Exploring face	MANOVA	Participants with 22q11.2DS spent more time looking at the mouth (*p* = 0.002)
(64)	21	14.86 (8–32)	–	Prodromal + schizophrenia family member + low risk	Identification of static facial expressions (labeling task)	Regression analysis	Individuals with 22q11.2DS had difficulties to label emotions (*p* < 0.0001)
(65)	63	13.7 (6–25)	80.5 (13.7)	22q11.2DS + TD	Ability to spontaneously attribute mental state (social-attribution task)	ANOVA	In comparison to controls, individuals with 22q11.2DS showed significant impairments in the ability to explain purposeful behavior (intentionality) (*p* < 0.001) and to describe accurately the events going on in the scene (appropriateness) (*p* < 0.01)
(25)	31	15.9	–	Healthy control	Cognitive ToM (TASIT task)	*T*-test	22qDS participants exhibited impaired performance on task of ToM (*p* < 0.001) and in recognition of lies (*p* < 0.001) and sarcasms (*p* < 0.001)
(66)	15	14.67 (9–19)	–	TD	Perception of facial expression (picture-to-picture matching task with morphed face stimuli)	ANOVA	In comparison with control, individuals with 22q11.2DS had more difficulties perceiving anger (*p* < 0.001), fear (*p* = 0.049) and sadness (*p* = 0.028) but not disgust (*p* = 0.21) and happiness (*p* = 0.11). They had difficulties to discriminate these facial emotions when they were moderately expressed, as frequently observed in daily life
(67)	17	17.44 (12–21)	72.11 (12.99)	TD	Identification of static facial expressions (labeling task)	ANOVA	The 22q11DS group had significantly more problems correctly identifying the emotions of anger (*p* = 0.0005), disgust (*p* = 0.002), fear (*p* = 0.001) and neutral (*p* = 0.009) They performed similarly to controls on facial emotions such as happy, surprise and sad (*p* > 0.05)
(68)	20	16.75 (8–21)	73.75 (13.63) [56–98]	Autism spectrum disorder (ASD) + TD	Identification of static facial expressions (labeling task) Exploring face	ANOVA	Participants with 22q11.2DS spent more time looking at the mouth compared to both the ASD and TD group (*p* = 0.02). No main effect of group or interaction were identified for the amount of time spent examining eye region
(69)	49	11	66.33 (8.6)	TD	Identification of static facial expressions (labeling task)	Mann–Whitney *U* tests	Individuals with 22q11.2DS had difficulties to label emotions (*p* < 0.001) in comparison with the TD group
(70)	60	10.5	–	TD	Vocal emotion recognition in stimuli with semantic message (paralanguage test)	Chi-square	No significant differences were found in the emotional perception and processing by paralanguage subtest between the two groups
(71)	29	15.7	79.52 [35–113]	TD	Identification of static facial expressions (labeling task with morphed image) Cognitive ToM (strange stories)	Wilcoxon rank-sum tests	Individuals with 22q11.2DS was significantly less accurate in detecting the sadness (*p* = 4.44 × 10^−7^), fear (*p* = 8.0 × 10^−4^), disgust (*p* = 6.77 × 10^−9)^, anger (*p* = 5.95 × 10^−7^), happiness (*p* = 5.0 × 10^−4^), sarcasm (*p* = 1.16 × 10^−6^), and sincere (*p* = 6.0 × 10^−4^) but not for the surprise (*p* = 0.3)

**Table 3:** Studies examining social cognition in Fragile X syndrome (FXS).

Reference	*N*	Mean age in years (range)	Full scale intelligence quotient (SD) [range]	Mental age (MA), verbal mental age (VMA), and non verbal mental age (NVMA) (SD) [range]	Comparison group	Social cognition domain (task)	Data analysis	Main results
(72)	30	20.93 (16.05–25.33)	67.78 (20.73) [40–119]	–	Comparison group (idiopathic developmental delay, intellectual disability, or learning disability)	Processing of egocentric gaze	ANOVA	Individual with FXS performed at the same level than patients with similar general cognitive abilities and autism symptoms (*p* = 0.53)
(73)	28	11:2 (7–15)	–	VMA 6.91 (1.75) [4.1–10.9]	Down syndrome (DS)	Ability to infer first-order false beliefs (Sally–Ann task and the appearance-reality task)	Chi-square test	Children with FXS and individuals with DS performed at a similar level During the Sally–Ann task (*p* = 0.77) and the appearance-reality task (*p* = 0.91)
(74)	22	47.91 (18–69)	105.18 (10.5)	–	Typically developing (TD) (from FXS families) + TD (from general population)	Identification of static facial expressions (labeling task) Identification of complex emotions (RMET)	ANOVA	In comparison with TD group, individuals with FXS were less proficient in discriminating basic emotion (*p* = 0.02) and complex emotion (*p* = 0.008)
(75)	13	19.70 (6.60–34.19)	–	–	Autism spectrum disorder (ASD)	Spontaneous perception of facial expression (oddball paradigms in conjunction with a measure of preferential looking)	*t*-test	Individuals with FXS or autism looked significantly longer at disgusted faces compared to neutral faces (FXS: *p* = 0.001; ASD: *p* = 0.001)
(76)	8	12.5 (10.25–14.16)	45.3 (3.2) [40–49]	VMA 6.58 (1.66) [4.83–9.50]	Intellectual disability of unknown etiology	Ability to infer first-order false beliefs (the Smarties task)	Chi-square test	Individuals with FXS were less accurate in the Smarties task (*p* < 0.05)
(77)	15	13.66	–	VMA: 6.11 (2.0) NVMA: 6.0 (1.3)	FXS-A + intellectual disability group	Ability to infer first-order false beliefs (Sally–Ann task)	ANOVA	Individuals with FXS or FXS-A were less accurate in the Sally–Ann task than the Intellectual Disability group (*p* = 0.009)
(78)	10	16.4 (9.7–24.0)	91 (16.2) [75–124]	–	TD	Identification of static facial expressions (labeling task)	*T*-test	Individuals with FXS were less proficient in discriminating neutral faces (*p* = 0.048), sadness (*p* = 0.070) and scrambled face (*p* = 0.016) than TD group
(79)	19	30.47 (18–40)	92.2 (15.3)	–	Obligate carrier + TD	Perception of facial expression (picture-to-picture matching task)	ANCOVA	Fragile X women do not demonstrate a deficit in emotion perception
(80)	16	24.8 (12.1–56.1)	64 (13.7) [51–96]	MA 8.4 (3.8) [6.0–21.1]	Typically developing matched on mental age (TDMA) + typically developing matched on chronological age (TDCA)	Identification of static facial expressions (labeling task)	ANOVA	FXS group performing significantly worse than both TDCA (*p* = 0.013) and TDMA (*p* = 0.044) group in their ability to recognize anger. They were also significantly worse at recognizing neutral expressions compared to TDMA group (*p* = 0.032)
(81)	15	41.80 (17–66)	36.60 (7.74)	–	Control	Identification of static facial expressions (labeling task)	ANOVA	No significant differences were found in the emotional perception and processing by paralanguage subtest between the two groups
(37) (first study)	14	10.34	–	VMA 5.72 (0.93) [3.9–6.9]	DS + TDMA	Perception of facial expression (picture-to-picture matching task)	ANOVA	No group difference in the ability to recognize emotion from facial expression (*p* = 0.110)
(37) (second study)	18	8.16 (4–14)	–	–	DS + intellectual disability of unknown etiology	Perception of facial expression (picture-to-picture matching task)	Fisher exact test	No group difference in the ability to recognize emotion from facial expression
(82)	13	15.5	61.0 (14.8)	–	Developmental delay (DD) + TDCA	Processing of egocentric gaze	MANOVA	Significant differences in task accuracy were revealed between the TD group and other groups (*p* = 0.02 and *p* = 0.03 vs. DD; *p* = 0.001 vs. FXS) but not between the DD and FXS groups (*p* = 0.99 for all)
(83)	12	20.6 (12.1–38.1)	64 (14.7) [52–96]	MA 9.0 (4.2) [6.1–21.1]	TDMA + TDCA	Identification of static facial expressions (labeling task)	ANOVA	FXS group performing significantly more poorly than both control groups when recognizing disgusted (TDMA *p* < 0.001; TDCA *p* = 0.001) and neutral (TDMA: *p* = 0.035; TDCA: *p* = 0.015) Facial expressions. TDCA group was significantly better at recognizing fearful facial expression compared to the FXS (*p* = 0.001) and (younger) TDMA (*p* = 0.001) groups
(84)	15	11.75	–	MA 4.08 (1.08)	DS + non-specific intellectual disability + TD	Perception of facial expression (picture-to-picture matching task)	ANOVA	No obvious deficit in basic emotion perception was reported in FXS group

**Table 4:** Studies examining social cognition in Turner syndrome (TS).

Reference	*N*	Mean age in years (range)	Full scale intelligence quotient (SD) [range]	Mental age (MA), verbal mental age (VMA), and non verbal mental age (NVMA) (SD) [range]	Comparison group	Social cognition domain (task)	Data analysis	Main results
(85)	26	30.58	–	–	Typically developing (TD)	Identification of static facial expressions (labeling task) Ability to spontaneously attribute mental state (social-attribution task)	*T*-test ANOVA	TS women are less accuracy in recognition of fear (*p* < 0.05) and sadness (*p* = 0.052) than TD group TS women being less accurate in describing the animations than TD women (*p* = 0.05)
(86)	51	25.1 (15–44)	–	–	Partial X chromosome deletion + TD	Identification of static facial expressions (labeling task)	MANOVA	Children and women with TS showed a specific impairment for the recognition of fearful (*p* < 0.0001) and angry faces (*p* < 0.006)
(87)	14	10.1 (6.92–12.92)	90.43 (12.74)	–	TD	Identification of static facial expressions (labeling task) Affective theory of mind (NEPSY-II)	ANOVA	Girls with TS were significantly less accurate in the classification of fearful faces compared with TD controls (*p* = 0.007), however, did not differ in accuracy for the other emotions (happy: *p* = 0.073, neutral: *p* = 0.106, scrambled: *p* = 0.179)
(88)	23	24.6 (18–36)	–	–	TD	Identification of static facial expressions (labeling task)	ANOVA	Fear and anger were significantly less well recognized by women with TS than controls: for fear (*p* < 0.01); for anger (*p* < 0.01)
(89)	18	32.6 (18–63)	104.8 (16.3)	–	TD	Identification of static facial expressions (labeling task)	MANOVA	The TS group were significantly impaired in recognizing fear relative to control females, *p* < 0.013. None of the other emotions showed significant difference between the two groups
(90)	40	25.44 (19–33)	90.68 (9.30) [75–106]	–	Noonan syndrome + TD	Identification of dynamic facial expression (labeling task)	ANOVA	The mixed between-within subjects ANOVA revealed no significant effect of Group, indicating that the three groups did not significantly differ in accuracy of emotion perception
(91)	71	27 (17–50)	96 (9)	–	TD	Identification of static facial expressions (labeling task)	ANOVA	In comparison with TD group, women with TS showed a specific impairment for the recognition of fear (*p* = 0.001) and angry (*p* = 0.001) faces
(92)	94	31.2	98 (10)	–	Women with premature ovarian failure + TD	Identification of static facial expressions (labeling task)	ANCOVA	The TS group had difficulty to recognize “anger” (*p* = 0.005), but no “happy,” “sad,” “fear,” “surprise,” and “disgust”

**Table 5:** Studies examining social cognition in Williams syndrome (WS).

Reference	*N*	Mean age in years (range)	Full scale intelligence quotient (SD) [range]	Mental age (MA), verbal mental age (VMA), and non verbal mental age (NVMA) (SD) [range]	Comparison group	Social cognition domain (task)	Data analysis	Main results
(93)	27	17.41 (5.33–43.66)	47 (19) [18–84]	MA 5.75 (1.1) [3.78–8.66]	Typically developing matched on mental age (TDMA) + typically developing matched on chronological age (TDCA)	Cognitive theory of mind (ToM)	ANOVA	Individuals with WS attributed less negative intentions than TDMA and TDCA groups (*p* < 0.001)
(57)	19	21.5 (7.16–38.83)	–	–	TDCA + TDVA	Identification of complex emotions (RMET)	*T*-test ANOVA, Tukey *post hoc* tests	Adults with WS perform at similar level than TDCA group when identifying whether the actors are “deciding” (*p* = 0.553), “not sure” (*p* = 0.279), or “worried” (*p* = 0.553) They were less accurate at identifying “ do not trust ” in comparison to TDCA group (*p* = 0.001) and TDVA group (*p* = 0.008), “disapproving” in comparison with TDCA group (*p* < 001), “relieved” in comparison to TDCA group (*p* = 0.013)
(94)	24	32.36 (15.4–56.9)	65 (7.10) [50–80]	–	Typically developing (TD)	Vocal emotion recognition in multisensory emotional information (paralanguage test)	*T*-test	Between-group differences in performance with the emotionally congruent multisensory (visual and vocal) did not reach the adjusted significance level (*p* = 0.07). But the TD group outperforming the WS group with the emotionally incongruent audiovisual stimuli (*p* < 0.001) and the unimodal auditory stimuli (*p* < 0.001)
(95)	12	11.4y (9.6–13.9)	–	–	Autism spectrum disorder (ASD) + TD	Vocal emotion recognition in stimuli without semantic message (paralanguage test)	ANCOVA	No between-group differences were found (*p* > 0.065)
(96)	8	27.8y (18–42)	–	–	DD + TD	Vocal emotion recognition in stimuli without semantic message (paralanguage test)	ANOVA *T*-test	The TD group outperforming the WS group (*p* = 0.004). The interaction effect was due to the WS group exhibiting lower recognition accuracy for negative stimuli (*p* = 0.001), while no between-group differences were in evidence for the processing of the positive stimuli (*p* =0.73)
(97)	21	24.0 (12–40)	–	–	DD + TD	Identification of static facial expressions (emotion is associated with congruent or non congruent evocativ music) Vocal emotion recognition in multisensory emotional information (paralanguage test)	ANOVA	Emotionally evocative and congruent music facilitated the ability of participants with WS, DD, or TD to process emotional facial expressions (*p* = 0.03)
(98)	57	9.24 (6.00–12.74)	72.93 (15.17) [40–97]	–	–	Ability to infer first-order false beliefs		WS patients had difficulties to understand false beliefs
(99)	12	8.10 (6.1–15.3)	–	VMA 5.8 (1.11) [3.4–9.3]	AUT (autism group) + TDVMA	Identification of static facial expressions (labeling task)	ANOVA	Individuals with WS showed emotion recognition levels similar to controls with the same mental age or individuals with developmental disabilities
(100)	25	9.5 (6–15)	54.7 (8.95)	–	–	Identification of static facial expressions (labeling task)		Individuals with WS recognized facial affect at an appropriate developmental level
(101)	20	12.25 (5.6–23.58)	–	MA 5.58 (0.75) [4.25–6.83]	DS + TDCA + TDMA	Identification of static facial expressions (labeling task)	ANOVA (pair-wise comparisons with Bonferroni correction)	In emotion recognition task, no significant differences were found between participants with WS and TDMA group but participants with WS performed significantly lower than TDCA group (*p* < 0.001). the DS group performed significantly lower than the WS (*p* ≤ 0.001)
(102)	11	30.6	66.4 (11.5)	–	TD	Processing of egocentric gaze	*T*-test	No difference in accuracy when WS and TD groups were compared (*p* < 0.057). However, WS participants were slower than controls (*p* < 0.0001) in determining the gaze direction
(103)	47	19.49 (12.1–32.4)	69.08 (12.2) [51–100]	–	Learning disability + TD	Identification of static facial expressions (labeling task) Vocal emotion recognition in stimuli with semantic message (paralanguage test)	ANCOVA	In labeling task and paralanguage test, WS group was significantly less accurate in recognition of sadness, anger and fear (*p* < 0.0001) than TD group. No difference is revealed between WS and learning disability groups
(104)	20	16.13 (5.33–43.67)	–	–	DS + TDCA + TDMA	Identification of static facial expressions (labeling task)	ANOVA	Individuals with WS showed emotion recognition levels similar to TDMA group (*p* > 0.1). WS groups outperformed the DS group (*p* < 0.05). And TDCA group outperformed WS group (*p* < 0.01)
(105)	31	17.02 (5.33–43.67)	–	MA 5.65 (1.31) [3.58–9.33]	TDCA + TDMA	Ability to infer first-order false beliefs (non-verbal picture-sequencing task)	ANOVA	The WS group performed significantly below the TDMA group on the false belief stories (*p* < 0.01). These groups performed similarly on all other story types (understanding of pretense and intent). TDCA group performed significantly better than the WS group and TDMA group on all story type (*p* < 0.01)
(106)	16	25.14 (11.42–50.58)	61 (15) [38–84]	–	TDMA	Identification of static facial expressions (labeling task) Exploring face	ANOVA	Individuals with WS showed emotion recognition levels similar to TDMA group: no significant main effect for group was revealed (*p* > 0.1) The WS group spent significantly more time looking at the eyes than TD controls (*p* < 0.05) but the groups spent a similar amount of time looking at the nose (*p* > 0.1) and the mouth (*p* = 0.1)
(107)	15	10.41 (6.0–15.83)	–	VMA7:2 (20)	ASD + TDVMA match with WS + TDNV match with WS + VMA match with ASD + TDNV match with ASD	Perception of facial expression (picture-to-picture matching task)	*T*-test	Participants with WS were more accurate when perceiving happiness, sadness, anger, and surprise relative to TDVMA (*p* < 0.05) and TDNV (*p* < 0.01) group, who did not differ
(108)	14	15.16 (8.75–28.0)	–	–	ASD + TDNV matched individually with WS + TDNV matched individually with ASD	Exploring face	*T*-test	Individuals with WS fixated faces for longer than participants who were developing typically (*p* < 0.001)
(109)	15	13.50 (8.66–28.0)	–	–	ASD + TDNV matched individually with WS + TDNV matched individually with ASD	Processing of allocentric gaze	*T*-test	In comparison with TDNV, participants with WS had difficulties to interpret eye-gaze direction (*p* < 0.05)
(110)	29	14.7 (7–27)	57.8 (12.3) [40–93]	MA 8.3 (2.9) [4–17]	TDMA +TDCA	Identification of static facial expressions (labeling task)	ANOVA	No such group differences were found for photographs of real faces (WS vs. TDMA, *p* > 0.05; WS vs. TDCA, *p* > 0.05)
(111)	19	14.4 (7–26)	57.5 (11.0)	MA 8.3 (3.5) VMA 9.3 (3.5) PMA 7.9 (2.7)	TD	Cognitive ToM (strange stories)	ANOVA (turkey test)	WS group were significantly less accurate on the visual than on the verbal modality (*p* < 0.001)
(112)	19	13.7 (5.1–30.0)	63.3 (12.33)	MA 6.5 (1.9) [4.1–11.2]	TDMA + TDCA	Processing of motor action	ANOVA	The WS and TDMA groups differed from performance of the TDCA group (*p* = 0.015 and *p* = 0.025, respectively). Most interestingly both the WS and the TDMA groups were significantly aided by the presence of contextual cues (*p* < 0.001), while the presence of contextual cueing brought no variation in performance within the TDCA group (*p* = 0.063)
(113)	16	12.58 (5.08–22.66)	64.9 (13.50) [44–87]	MA 6.5 (1.33) [4.1–11.2]	ASD + TDMA + TDCA	Processing of motor action	ANOVA	In the presence of context cues, children with WS were as accurate as children with the same chronological age and children with the same mental age in determining why others perform specific motor actions. Amount of errors made by all groups did not differ
(114)	29	19.1 (13.1–32.1)	68.1 (12.8) [45–94]		Learning/intellectual disability + TDCA	Identification of dynamic facial expression (labeling task)	ANOVA	Participants with WS were less accurate than controls with TDCA (*p* < 0.001). But they were as accurate as participants with intellectual disabilities (*p* = 0.87)
(115)	21	7.16 (4.5–8.58)	68 (12) [43–93]	VMA 4.91 (1.33) [3.1–8.2]	Prader–Willi syndrome (PWS) + NMR	Perception of facial expression (picture-to-picture matching task) Ability to infer first-order false beliefs (similar to the Sally–Ann task) Affective ToM (explanation of action task)	Chi-square test	On the false belief question, more of the MRU and PWS children passed than did the WMS children (*p* < 0.06). The three groups performed at a similar level in picture-to-picture matching test and the explanation of action task
(116)	30	9.91 (5.00–17.08)	–	–	TD	Ability to infer first-order false beliefs (task similar to the Sally–Ann task but presented in video and without narrative)	Fisher’s exact test	More participants in the WS group failed the false belief question in contrast to the TD group (*p* = 0.001)

## Results

The large majority of studies assessed either emotion recognition or ToM whereas fewer studies documented attributional style and social knowledge. Moreover, in some area of social cognition, very few studies or no study at all were available for some syndromes, especially AS, RS, and SMS.

### Emotional Information Processing

#### Recognition of Facial Emotion

Basic emotions defined by Ekman (14), namely joy, sadness, anger, fear, and surprise, are the first emotions recognized in faces by children with typical development (117). No obvious deficits in basic emotion perception were reported in females with FXS (37, 79, 81, 84). However, males with FXS looked significantly longer at disgusted faces compared to neutral faces (75), with more difficulties to recognize sadness and fear (83). The same pattern of results was revealed in children with ASD. By contrast, individuals with RS spend less time than controls exploring key facial features (eye, nose, and mouth) for all emotions (118) and have difficulties to recognize emotional expressions (happiness, fear, and sadness) in general. These results may be due to increased attentional or informative value of negative over neutral (and positive) expressions (75).

People with 22q11.2DS have difficulties to discriminate facial emotions when they are moderately expressed (66), which can explain major deficits in social interactions, especially during childhood. Moreover, they have more difficulties to perceive negative emotions such as anger, fear, and sadness and to label emotions (64, 69, 71) while happiness, sadness, and surprise recognition seems efficient (59, 67, 68). A recent study using the CANTAB ERT test (morphed images with fast cover up times to avoid ceiling effects) showed that 22q11.2DS patients are significantly less accurate in detecting sadness and happiness (71). The same results are observed in males with FXS (73, 74, 78, 80, 83, 119).

Patients with WS showed emotion recognition levels similar to controls with the same mental age or individuals with developmental disabilities (99–101, 104, 106, 107, 110, 114). They are more accurate in perceiving happiness, sadness, anger, and surprise (107) but perform more poorly on auditive stimuli for all emotions except for happiness, which seems relatively surprising considering the classical hyperacusis associated with the syndrome (103). Abnormalities in auditory processing in WS seem to be restricted to the perception of negative affective vocalizations, such as scream or gasp (96). However, children with WS had no difficulty to recognize vocal emotions compared to controls (94, 95). Emotional information can be transmitted *via* both visual (e.g., facial expressions and gestures) and auditory (e.g., affective prosody) channels. In social interactions, both channels are competing and require the integration of relevant multisensory emotional information. When identifying facial affects, patients with WS are as accurate as controls in both congruent and incongruent conditions. But they are less efficient when identifying non-social affects (97). These results confirmed that emotionally evocative music facilitates the ability of patients with WS to recognize emotional faces. They spend more time staring at the salient features of the face—particularly the eyes (106, 108). Interestingly, the ability to discriminate and match faces with emotions was assessed in children with WS syndrome compared to PWS syndrome, or a non-specific intellectual deficiency. The three matched groups performed at a similar level (115) without significant correlation between chronological age (CA) and performance on face perception tasks (107). Overall, neurocognitive deficits do not seem to be the only explanation of expression recognition impairments across syndromes, as illustrated in TS (88).

Adults with WS demonstrated a normal level of performance in the identification of emotions in unimodal conditions with face stimuli alone and in congruent face-voice multisensory contexts. However, they are less accurate to identify emotions in unimodal conditions with vocal emotions alone and even worse in multisensory incongruent stimuli conditions (95, 98). This difference seems to be highly specific and central to understand the hypersociability of WS (Table 1). Patients with WS may be specifically attracted by facial information, decreasing their attention to vocal information. Interestingly, when adults with WS are asked to identify complex mental states (such as worry, disinterest, etc.) from the whole dynamic face, their performance is similar to controls matched on CA (57). However, they have difficulties with “relief” and “distrust,” which may be related to hypersociability.

The deficit is more pronounced in PWS and WS, with difficulties to recognize all basic emotions (8, 99, 101). A global emotion identification impairment was documented in 22q11.2DS, FXS, PWS, and WS but the recognition of happiness seems preserved in these syndromes (101). Regarding the recognition of complex emotions, patients with PWS are less efficient than intelligence quotient (IQ)-matched individuals with WS. This result is consistent with the distinct behavioral symptoms associated to these syndromes (complex psychological disorder with maladaptive behaviors vs. frequent hypersociability).

During a task assessing the recognition of anger, fear, disgust, or sadness, individuals with KS were less accurate in recognizing angry faces, unlike other facial expressions (120). Children and women with TS show a specific impairment for the recognition of fearful and angry faces, both when the stimulus is a whole face or the upper face only (85–89, 91, 92). More specifically, in TS, fear recognition is significantly weaker compared to other emotions, including anger (88), with difficulties to correctly identify affect tones (91). In a study using dynamic facial expressions of different emotional intensities, patients with TS, 22q11.2DS and controls did not show any deficit in recognizing fear (62, 90). But patients with 22q11.2 are slower to recognize emotions (62) with a normal level of emotion recognition in auditory stimuli (70). By contrast, dynamic stimuli in WS do not change the accuracy of emotion recognition (114).

During an emotion recognition task using photographs, patients with 22q11.2 (59) spent less time than controls observing the eye region, as observed in FXS and ASD. They spent more time looking at the mouth (63, 68), which may explain why they have less difficulty to recognize happiness, surprise and, to a lesser extent, disgust. This result is correlated with the high prevalence of ASD in 22q11.2DS and FXS. By contrast, women with TS stare longer at the mouth, but only when looking at fearful faces. This result suggests a possible mechanism for selective fear deficit (89) across syndromes. Facial emotion recognition impairment may be related to an atypical observation of faces (121). Fewer fixations on the eyes region are related to difficulties to recognize specific emotions. This deficit is a core symptom in ASD controls who stare longer at the mouth for joy, fear, and disgust; at the eye for sadness; and at the eye and nose for anger (122).

### ToM in Developmental Syndromes With Psychiatric Phenotype

#### Ability to Infer False-Belief in Others

An important mechanism of ToM is the ability to understand that other people’s beliefs or representations about the world may differ from reality.

Children with 22q11.2 did not show major difficulties in ToM tasks (60). When it comes to PWS and WS, children had difficulties attributing mental states and understanding first-order beliefs (98, 116, 123). These results were confirmed in a non-verbal picture-sequencing task, but with a preservation of the understanding of intention, social script knowledge, and physical cause-and-effect reasoning (105). These results suggest that language abilities do not influence performance on ToM in WS (116), considering the heterogeneity of the process in the syndrome (105).

Patients with FXS are less accurate in ToM tasks (76, 77) and perform at a similar level than individuals with Down syndrome (DS) (73, 74) or ASD (77), with a limited impact of autistic symptoms (124). Altogether, these findings suggest that a ToM disorder in FXS may not result solely from the high comorbidity of ASD (77).

Children with WS, PWS, or a non-specific intellectual deficiency, perform at a similar level in standard ToM tasks (115, 125). However, children with PWS had specific difficulties to attribute second-order beliefs (123, 126).

Interestingly, 22q11.2DS seems specifically associated with a poor performance on false-belief tasks (60, 61), this deficit being highly correlated with psychotic symptoms.

#### Ability to Infer Mental States in Others

Children with 22q11.2 seem to have difficulties to link contextual and social relevant cues when the scene is visually complex (61) or based on circumstances (61). This deficit may be related to attentional and visuospatial disorders and requires families and therapists to clearly verbalize emotions during social interactions. Moreover, the comprehension of misunderstanding, persuasion, pretending, sarcasm, and white lie seems impaired in 22q11.2DS (25, 60, 71). These findings suggest that a cognitive ToM disorder causes a delay rather than a deficit (60). From this perspective, impairments in the spontaneous attribution of seducing, mocking, playing with one another, getting frightened, or elated mental states to abstract visual stimuli were reported in 22q11.2DS (65), suggesting that gaze direction is also a weakness in the syndrome (60).

As expected, patients with WS were proficient during a verbal task (111, 126) but their performance decreased when the ToM task was presented on a visual medium (111) with difficulties inferring mental states from visual cues, especially for negative intentions (93) and gaze direction (109). These results suggest that they may present a positive bias in the interpretation of social cues. An impaired social-perceptual ability may play a role in increased approachability and a deficit in the processing of basic social cues may have possible repercussions on ToM. The ability to observe and recognize other people’s actions contributes to understand goals and intentions (127) and may help people with WS improve their recognition of action process (113). Indeed, in the presence of context cues, children with WS were as accurate as controls (112, 113).

Overall, patients with 22q11.2DS, FXS, or WS had difficulties determining whether someone is looking at them or elsewhere (60, 82, 102, 128) but individuals with PWS seem to yield scores within the normal range (129). When patients with FXS were asked to judge the direction of eye gazes (direct or averted), they performed at the same level than patients with similar general cognitive abilities and autism symptoms (72), and patients with a developmental delay (82). Complex emotion recognition requires knowledge and analysis of interpersonal relationships, unlike basic emotion recognition. Thus, further research on the potential dissociation between the recognition of complex mental states and basic emotions, and the influence of the nature of the stimuli (static or dynamic) is needed.

## Discussion

### Interventions and Perspectives

This systematic review shows that social cognition deficits are present in neurodevelopmental disorders at different levels and remain strongly correlated with psychiatric phenotype. Emotion recognition and ToM skills impairments seem to be a core deficit in rare developmental syndromes, otherwise associated with psychosis and ASD. Although more research needs to be done to assess social cognition phenotypes in these syndromes, it is crucial to develop and evaluate appropriate therapeutic interventions. In this section, we briefly discuss therapeutic approaches and present future keys to improve social deficits in that indication.

In daily life, facial expressions are typically embedded in a rich context with many distractors (e.g., noises and visual stimuli) and social information from multiple sensory channels (e.g., prosody, gestures, and change in facial expression). Therefore, social interactions necessitate fast and efficient cognitive skills. They include the ability to identify visual and spatial relationships in emotion recognition, to integrate and manipulate such perceptions, to select relevant information, to inhibit irrelevant stimuli and alternate between several sensitive channels. Emotion recognition is also a complex process involving visual attention (130), visuospatial abilities (130), working memory (131), divided attention and executive functions (132). As a consequence, improvement of these functions may have a positive impact on social cognition and behavior. Visuospatial and visuoperceptual skills play a key role in everyday life. Visual information and complex visual stimuli are analyzed with a complete unawareness of the visuoperceptual process or the complexities of the stimuli involved. This process becomes conscious in a context of learning. Repetition and familiarity enable a more spontaneous approach and turn the conscious and effortful process into an automatic one. If this ability is impaired, many types of deficits can occur, ranking from a failure to process the basic elements of a visual stimulus (i.e., colors, lines, and orientation) to more complex and integrative features, such as object identification, faces, or familiar scenes. These deficits can include social cognition defects, especially in the area of facial emotion recognition, acquired slowly during childhood and reach adult levels in late adolescence (133).

Currently, two programs, named “Cognitus & Moi” (Cognitus & Me) and “Vis-à-Vis” have considered the link between neuropsychological functions and social cognition (facial emotion recognition). “Cognitus & Moi” is a cognitive remediation program with SCT, designed for 5- to 13-year-old children with developmental disorders. The program is specifically focused on attentional, visuospatial functions and emotion recognition (134). It involves a variety of exercises in a paper and/or pencil (*n* = 30) or a computerized format (*n* = 29) and a strategy coaching approach. “Vis-à-Vis” is a computerized training program based on a trial-and-error approach. It can be performed at home with parents and targets social cognition difficulties and working memory (135).

The ability to explore face efficiently develops during childhood. The eyes, nose, and mouth are the preferred attentional targets in facial exploration (121). These facial features play an important role in perception and recognition of emotion. However, the visual exploration developed by individuals with neurodevelopmental syndrome is mainly different, with a lack of eye contact according to a specific pattern previously observed in ASD. Individuals with neurodevelopmental syndrome may benefit from educational solutions targeting this impairment. In that regard, an important point is that dynamic information facilitates the perception of facial expressions (136, 137). Individuals with autism appear to benefit from slow dynamic information when categorizing emotional expressions (138). Considering the possibility of overlap between children with neurogenetic disorders and children with typical ASD (139), the question of a similar benefit in rare diseases should also be addressed in the future. More generally, the beneficial effect of motion may play a role in therapeutic intervention.

Some current treatment plans which are based on phenotype rather than etiology may be adapted for individual with neurodevelopmental syndrome.

For example, considering the well-established association between 22q11.2DS and schizophrenia, SCT, a program based on the cognitive enhancement therapy developed for individuals with schizophrenia (140) may be of interest in 22q11.2DS. This program targets general and high-level social skills such as the ability to practice calming techniques, to identify the main idea in a conversation, to determine expected behaviors in different situations (social perception and social knowledge), or to clearly express one’s own thoughts (ToM) (140).

It is necessary to define strengths and weaknesses of each participant and the exact nature of deficits. Indeed this review highlights the implication of different cognitive processes involved in social interaction deficits, which appear to have different effects across syndromes (Tables 6 and 7). Here, we show that social cognition impairments are not uniquely caused by syndrome comorbidities such as IQ. Understanding the differences in the social cognitive abilities may become a distinctive clinical tool to develop special trainings. In several cases, the severe cognitive impairment associated with rare diseases necessitates individual adaptation of therapeutic interventions. Individual may present severe limitations in their ability to communicate through speech and gestures. Single case reports have demonstrated that patients can develop communicative skills and social cognition, particularly affective and cognitive ToM with appropriate interventions. For example, communication by composing words on an alphabetic table may be improved by training communicative skills in RS (141). With a training focused on emotion, discrimination of facial emotion (joy, sadness, and anger) may be improved in RS (142). Moreover, although patients do not develop functional verbal skills, the eye-tracking technology has increased the possibilities to understand patients (143) and facilitated the access to augmentative and alternative communication (144–146).

**Table 6:** Emotion processing among rare neurodevelopmental syndrome.

	22q11.2DS	Fragile X syndrome	Klinefelter syndrome	Prader–Willi syndrome	Rett syndrome	Turner syndrome	Williams syndrome
Basic facial emotion perception	Deficit in anger, fear, and sadness perception in comparison with typically developing matched on chronological age (TDCA) participants. No deficit in disgust and happiness perception	No obvious deficit in basis facial emotion recognition in comparison with TDCA participants		Not deficit in basis facial emotion perception compared to participants with WS			Not deficit in happiness, sadness, anger. and surprise perception [typically developing matched on mental age (TDMA)]
Spontaneous perception of facial expression		Not deficit in happiness and disgust perception in comparison with typically developing (TD) participants			Deficit in happiness, sadness, and fear perception in comparison with TDCA participants		
Basic static facial emotion identification	Deficit in fear, anger, disgust happiness, and sadness identification in comparison with TDCA participants	Deficit in anger, sadness, and disgust identification in comparison with TDCA participants	Specific deficit in anger identification	Deficit in all basic emotion identification in comparison with TDCA participants		Specific deficit in fear and anger identification	Deficit in all basic emotion identification in comparison with TDCA participants; no deficit in basis facial emotion identification in comparison with TDMA participants
Basic dynamic facial emotion identification	Not deficit in basic dynamic facial emotion identification in comparison with TDCA participants					Not deficit in fear identification	Deficit in all basic emotion identification in comparison with TDCA participants
Complex facial emotion identification	Deficit in complex facial emotion identification (TD)	Deficit in complex facial emotion identification (TD)		Deficit in complex facial emotion identification (WS)			Deficit in static complex facial emotion identification but no deficit in dynamic facial emotion (“deciding,” “not sure,” and “worried”) identification in comparison with TDCA participants
Emotion recognition in auditory stimuli	Not deficit emotion recognition in auditory stimuli in comparison with TDCA participants					Deficit emotion recognition in auditory stimuli in comparison with TDCA participants	Deficit in all emotion recognition except happiness in comparison with TDCA participants

**Table 7:** Theory of mind among rare neurodevelopmental syndrome.

	22q11.2DS	Fragile X	Prader–Willi	Turner	Williams
Ability to infer first-order false-belief	Deficit in first-order false-belief inferring in comparison with typically developing matched on chronological age (TDCA) participants	No deficit in first-order false-belief inferring compared to participant with DS	Deficit in first-order false-belief inferring in comparison with TDCA participants but no deficit compared to participants with WS or non-specific mental retardation		Deficit in first-order false-belief inferring in comparison with TDCA participants, but no deficit compared to participants with Prader–Willi syndrome (PWS) or non-specific mental retardation
Ability to infer second-order false beliefs	Deficit in second-order false-belief inferring (for only younger participants) in comparison with TDCA participants		Deficit in second-order false-belief inferring in comparison with TDCA participants but no deficit compared to participants with WS or non-specific mental retardation		Deficit in second-order false-belief inferring in comparison with TDCA but no deficit compared to participants with PWS or non-specific mental retardation
Affective theory of mind (ToM)	Deficit in affective ToM in comparison with TDCA participants		No deficit in ToM affective compared to participants with WS or non-specific mental retardation	Deficit in affective ToM in comparison with TDCA participants	No deficit in affective ToM compared to participant with PWS or Nons specific mental retardation
Cognitive ToM	Deficit for only younger participants in cognitive ToM in comparison with TDCA participants		Deficit in cognitive ToM in comparison with TDCA participants		Not deficit for a verbal task [typically developing (TD)] but deficit with a visual task (TD)
Ability to spontaneously attribute mental state	Spontaneously attribute mental state disability in comparison with TDCA participants			Spontaneously attribute mental state disability in comparison with TDCA participants	Spontaneously attribute mental state disability (not comparison group)

Face recognition and ToM have an important role in social problem solving (14, 147, 148). These capacities allow to understand and interpret other’s emotion (149). In the social interaction, recognition of expressions leads to adapt behavior (150, 151).

### Limitations

This systematic review highlights some limits. Some rare syndromes received only little interest, particularly AS, KS, RS, and SMS. The lack of results regarding some areas of social cognition in these syndromes must be interpreted with caution, as the number of published studies is limited. Furthermore, very few studies have directly compared patients considering anxiety levels. Moreover, psychiatric diagnosis has not been considered and very few studies have proposed to establish a link between social cognition and behavior in rare neurodevelopmental syndromes. In fact, the psychiatric phenotype has not yet been extensively studied. Many data are available for facial emotion recognition and ToM, but a lack of research in social perception/knowledge and attributional style has been highlighted. Thus, it remains unclear whether individuals with a rare developmental syndrome show similar impairments in these areas. Moreover, impairments may be specific to tasks and emotions, rather than all social cognitive processes. The use of relatively consistent methodology across study designs would help compare these impairments.

### Summary and Conclusion

To sum up, some social trainings, such as the use of the eye-tracking technology in RS or the focus on visuospatial functions in 22q11.2DS, are created among the specific profile of individuals with a neurodevelopmental syndrome. Understanding the differences in the social cognitive abilities of people with rare neurodevelopmental disorders may lead to a more personalized medicine. This review suggests that cognitive remediation therapy and SCT may be a viable way to improve the social and functional outcomes, although many questions on its effectiveness still need to be answered.

The studies targeting social cognition processes offer new thoughts about the development of specific cognitive training programs, as they highlight the importance of connecting neurocognitive and social cognitive training techniques. They point out that individuals with social cognitive impairments may need more help in terms of social interactions. They provide keys for caregivers, who may better adjust their communication with explicit social cues by mentioning what they are thinking or feeling and why. Understanding and adjusting attitudes to individuals with social cognition impairments are required to prevent frustration and maladaptive behaviors.

## Availability of Data and Supporting Materials Section

Data sharing not applicable to this article as no datasets were generated or analyzed during the current study.

## Author Contributions

AM elaborated the PRISMA review. AM and CD wrote the paper and collected the data. EP, AL, EF, and NF collected the data. All authors approved the final version of the manuscript.

## Conflict of Interest Statement

The authors declare that the research was conducted in the absence of any commercial or financial relationships that could be construed as a potential conflict of interest.

## Abbreviations

22q11.2DS, 22q11.2 deletion syndrome; AAC, augmentative and alternative communication; ASD, Autism spectrum disorder; AS, Angelman syndrome; CA, chronological age; DS, Down syndrome; FXS, Fragile X syndrome; IQ, intelligence quotient; KS, Klinefelter Syndrome; MA, Mental Age; NA, non available; PWS, Prader–Willi syndrome; RS, Rett Syndrome; SMS, Smith–Magenis syndrome; TD, typically developing; TDCA, typically developing matched on chronological age; TDMA, typically developing matched on mental age; ToM, theory of mind; TS, Turner syndrome; VIQ, verbal intelligence quotient; VMA, Verbal Mental Age; WS, Williams syndrome.

## References

[1] Crespi B. Genomic imprinting in the development and evolution of psychotic spectrum conditions. Biol Rev Camb Philos Soc (2008) 83(4):441–93. 10.1111/j.1469-185X.2008.00050.x 18783362

[2] Crespi B Badcock C. Psychosis and autism as diametrical disorders of the social brain. Behav Brain Sci (2008) 31(3):241–60. 10.1017/S0140525X08004214 18578904

[3] Crespi B Summers K Dorus S. Genomic sister-disorders of neurodevelopment: an evolutionary approach. Evol Appl (2009) 2(1):81–100. 10.1111/j.1752-4571.2008.00056.x 25567849 PMC3352408

[4] Norkett EM Lincoln SH Gonzalez-Heydrich J D’Angelo EJ Social cognitive impairment in 22q11 deletion syndrome: A review. Psychiatry Res (2017) 253:99–106. 10.1016/j.psychres.2017.01.103 28364592

[5] Biswas AB Furnis F Cognitive phenotype and psychiatric disorder in 22q11.2 deletion syndrome: A review. Res Dev Disabil (2016) 53(54):242–57. 10.1016/j.ridd.2016.02.010 26942704

[6] Swillen A The importance of understanding cognitive trajectories: the case of 22q11.2 deletion syndrome. Curr Opin Psychiatry (2016) 29(2):133–7. 10.1097/YCO.0000000000000231 26779858 PMC5414032

[7] Bostrom C Yau SY Majaess N Vetrici M Gil-Mohapel J Christie BR Hippocampal dysfunction and cognitive impairment in Fragile-X Syndrome. Neurosci Biobehav Rev (2016) 68:563–74. 10.1016/j.neubiorev.2016.06.033 27345143

[8] Whittington J Holland T Recognition of emotion in facial expression by people with Parder-Willi syndrome. J Intellect Disabil Res (2011) 55(1):75–84. 10.1111/j.1365-2788.2010.01348.x 21121995

[9] Rice LJ Einfeld SL Cognitive and behavioural aspects of Prader-Willi syndrome. Curr Opin Psychiatry (2015) 28(2):102–6. 10.1097/YCO.0000000000000135 25599341

[10] Poisson A Nicolas A Cochat P Sanlaville D Rigard C de Leersnyder H Behavioral disturbance and treatment strategies in Smith-Magenis syndrome. Orphanet J Rare Dis (2015) 4(10):111 10.1186/s13023-015-0330-x PMC4559928 26336863

[11] Fisher MH Morin L Addressing social skills deficits in adults with Williams syndrome. Res Dev Disabil (2017) 71:77–87. 10.1016/j.ridd.2017.10.008 29032288

[12] Green MF Olivier B Crawley JN Penn DL Silverstein S. Social cognition in schizophrenia: recommendations from the measurement and treatment research to improve cognition in schizophrenia. New approaches conference. Schizophr Bull (2005) 31(4):882–7. 10.1093/schbul/sbi049 16135561

[13] Elfenbein HA Ambady N. On the universality and cultural specificity of emotion recognition: a meta-analysis. Psychol Bull (2002) 128(2):203–35. 10.1037/0033-2909.128.2.203 11931516

[14] Ekman P Universal and Cultural Differences in Facial Expression of Emotion. Lincoln, NB: Nebraska University Press (1972).

[15] Calder AJ Young AW Perrett DI Etcoff NL Rowland D Categorical perception of morphed facial expressions. Vis cogn (1996) 3(2):81–117. 10.1080/713756735

[16] Baron-Cohen S Mindblindness: An Essay on Autism and Theory of Mind. Cambridge, MA: MIT Press (1995).

[17] Premack D Woodruff G Does the chimpanzee have a theory of mind? Behav Brain Sci (1978) 1(4):515–26. 10.1017/S0140525X00076512

[18] Wimmer H Perner J Beliefs about beliefs: representation and constraining function of wrong beliefs in young children’s understanding of deception. Cognition (1983) 13(1):103–28. 10.1016/0010-0277(83)90004-5 6681741

[19] Kalbe E Schlegel M Sack AT Nowak DA Dafotakis M Bangard C Dissociating cognitive from affective theory of mind: a TMS study. Cortex (2010) 46(6):769–80. 10.1016/j.cortex.2009.07.010 19709653

[20] Shamay-Tsoory SG Aharon-Peretz J. Dissociable prefrontal networks for cognitive and affective theory of mind: a lesion study. Neuropsychologia (2007) 45(13):3054–67. 10.1016/j.neuropsychologia.2007.05.021 17640690

[21] Fiske ST Taylor SE Social Cognition. 2nd ed New York: McGraw-Hill Book Co (1991).

[22] Oskarsdottir S Vujic M Fasth A. Incidence and prevalence of the 22q11 deletion syndrome: a population-based study in Western Sweden. Arch Dis Child (2004) 89(2):148–51. 10.1136/adc.2003.026880 14736631 PMC1719787

[23] Shprintzen RJ Velo-cardio-facial syndrome: 30 years of study. Dev Disabil Res Rev (2008) 14(1):3–10. 10.1002/ddrr.2 18636631 PMC2805186

[24] Angkustsiri K Goodlin-Jones B Deprey L Brahmbhatt K Harris S Simon TJ Social impairments in chromosome 22q11.2 deletion syndrome (22q11.2DS): autism spectrum disorder or a different endophenotype? J Autism Dev Disord (2014) 44(4):739–46. 10.1007/s10803-013-1920-x 24045981 PMC4327991

[25] Jalbrzikowski M Carter C Senturk D Chow C Hopkins JM Green MF Social cognition in 22q11.2 microdeletion syndrome: relevance to psychosis. Schizophr Res (2012) 142(1–3):99–107. 10.1016/j.schres.2012.10.007 23122739 PMC3714207

[26] Debbané M Glaser B David MK Feinstein C Eliez S. Psychotic symptoms in children and adolescents with 22q11.2 deletion syndrome: neuropsychological and behavioral implications. Schizophr Res (2006) 84(2–3):187–93. 10.1016/j.schres.2006.01.019 16545541

[27] Buckley RH Dinno N Weber P. Angelman syndrome: are the estimates too low? Am J Med Genet (1998) 80(4):385–90. 10.1002/(SICI)1096-8628(19981204)80:4<385::AID-AJMG15>3.0.CO;2-9 9856568

[28] Petersen MB Brondum-Nielsen K Hansen LK Wulff K Clinical, cytogenetic, and molecular diagnosis of Angelman syndrome: estimated prevalence rate in a Danish county. Am J Med Genet (1995) 60(3):261–2. 10.1002/ajmg.1320600317 7573182

[29] Mount R Oliver C Berg K Horsler K Effects of adult familiarity on social approach behaviours in Angelman syndrome. J Intellect Disabil Res (2011) 55:339–50. 10.1111/j.1365-2788.2010.01364.x 21255175

[30] Adams D Horsler K Oliver C Age related change in social behaviour in children with Angelman syndrome. Am J Med Genet A (2011) 155:1290–7. 10.1002/ajmg.a.33964 21567915

[31] Walz NC. Parent report of stereotyped behaviors, social interaction, and developmental disturbances in individuals with Angelman syndrome. J Autism Dev Disord (2007) 37:940–7. 10.1007/s10803-006-0233-8 17019625

[32] Steffenburg S Gillberg CL Steffenburg U Kyllerman M. Autism in Angelman syndrome: a population-based study. Pediatr Neurol (1996) 14:131–6. 10.1016/0887-8994(96)00011-2 8703225

[33] Crawford DC Acuña JM Sherman SL. FMR1 and the Fragile X syndrome: human genome epidemiology review. Genet Med (2001) 3(5):359–71. 10.1097/00125817-200109000-00006 11545690 PMC4493892

[34] Hall SS Frank MC Pusiol GT Farzin F Lightbody AA Reiss AL. Quantifying Naturalistic social gaze in Fragile X syndrome using a novel eye tracking paradigm. Am J Med Genet B Neuropsychiatr Genet (2015) 168(7):564–72. 10.1002/ajmg.b.32331 26079280 PMC5759950

[35] Murphy MM Abbeduto L Schroeder S Serlin R. Contribution of social and information-processing factors to eye-gaze avoidance in Fragile X syndrome. Am J Ment Retard (2007) 112:349–60. 10.1352/0895-8017(2007)112[0349:COSAIF]2.0.CO;2 17676959

[36] Baumgardner TL Reiss AL Freund LS Abrams MT. Specification of the neurobehavioral phenotype in males with Fragile X syndrome. Paediatrics (1995) 5:744–52. 7724315

[37] Turk J Cornish K Face recognition and emotional perception in boys with Fragile X syndrome. J Intellect Disabil Res (1998) 42(6):490–9. 10.1046/j.1365-2788.1998.4260490.x 10030445

[38] Bojesen A Juul S Gravholt C. Prenatal and postnatal prevalence of Klinefelter syndrome: a national registry study. J Clin Endocrinol Metab (2003) 88(2):622–6. 10.1210/jc.2002-021491 12574191

[39] Bishop DV Jacobs PA Lachlan K Wellesley D Barnicoat A Boyd PA Autism, language and communication in children with sex chromosome trisomies. Arch Dis Child (2011) 96:954–9. 10.1136/adc.2009.179747 20656736 PMC3182523

[40] Cordeiro L Tartaglia N Roeltgen D Ross J. Social deficits in male children and adolescents with sex chromosome aneuploidy: a comparison of XXY, XYY, and XXYY syndromes. Res Dev Disabil (2012) 33:1254–63. 10.1016/j.ridd.2012.02.013 22502852 PMC3328784

[41] Whittington J Holland A Webb T Butler J Clarke D Boer H Population prevalence and estimated birth incidence and mortality rate for people with Prader-Willi syndrome in one UK health region. J Med Genet (2001) 38(11):792–8. 10.1136/jmg.38.11.792 11732491 PMC1734766

[42] Kerr AM Witt Engerström I The clinical background to the Rett disorder. In: Ingegerd WE Kerr AM, editors. Rett Disorder and the Developing Brain. Oxford: Oxford University Press (2001). p. 1–26.

[43] Bartl-Pokorny KD Marschik PB Sigafoos J Tager-Flusberg H Kaufmann WE Grossmann T Early socio-communicative forms and functions in typical Rett syndrome. Res Dev Disabil (2013) 34:3133–8. 10.1016/j.ridd.2013.06.040 23891731 PMC5951273

[44] Didden R Korzilius H Smeets E Green VA Lang R Lancioni GE Communication in individuals with Rett syndrome: an assessment of forms and functions. J Dev Phys Disabil (2010) 22:105–18. 10.1007/s10882-009-9168-2 20339577 PMC2837828

[45] Urbanowicz A Leonard H Girdler S Ciccone N Downs J. Parental perspectives on the communication abilities of their daughters with Rett syndrome. Dev Neurorehabil (2016) 19:17–25. 10.3109/17518423.2013.879940 24564222

[46] Juyal RC Figuera LE Hauge X Elsea SH Lupski JR Greenberg F Molecular analyses of 17p11.2 deletions in 62 Smith-Magenis syndrome patients. Am J Hum Genet (1996) 58(5):998–1007. 8651284 PMC1914618

[47] Martin SC Wolters PL Smith ACM. Adaptive and maladaptive behavior in children with Smith-Magenis syndrome. J Autism Dev Disord (2006) 36:541–52. 10.1007/s10803-006-0093-2 16570214

[48] Sarimski K Communicative competence and behavioral phenotype in children with Smith–Magenis syndrome. Genet Couns (2004) 15:347–55. 15517828

[49] Stochholm K Juul S Juel K Naeraa RW Gravholt CH. Prevalence, incidence, diagnostic delay, and mortality in Turner syndrome. J Clin Endocrinol Metab (2006) 91(10):3897–902. 10.1210/jc.2006-0558 16849410

[50] Lagrou K Froidecoeur C Verlinde F Craen M De Schepper J François I Psychosocial functioning, selfperception and body image and their auxologic correlates in growth hormone and oestrogen-treated young adult women with Turner syndrome. Horm Res (2006) 66:277–84. 10.1159/000095547 16946621

[51] McCauley E Feuillan P Kushner H Ross JL. Psychosocial development in adolescents with Turner syndrome. J Dev Behav Pediatr (2001) 22:360–5. 10.1097/00004703-200112000-00003 11773800

[52] Hong DS Dunkin B Reiss AL. Psychosocial functioning and social cognitive processing in girls with Turner syndrome. J Dev Behav Pediatr (2011) 32:512–20. 10.1097/DBP.0b013e3182255301 21743350 PMC3179767

[53] Lepage JF Dunkin B Hong DS Reiss AL. Impact of cognitive profile on social functioning in prepubescent females with Turner syndrome. Child Neuropsychol (2013) 19:161–72. 10.1080/09297049.2011.647900 22372383 PMC3485432

[54] Suzigan LZ de Paiva e Silva RB Guerra-Júnior G Marini SH Maciel-Guerra AT. Social skills in women with Turner syndrome. Scand J Psychol (2011) 52:440–7. 10.1111/j.1467-9450.2011.00887.x 21534980

[55] Strømme P Bjornstad PG Ramstad K. Prevalence estimation of Williams syndrome. J Child Neurol (2002) 17(4):269–71. 10.1177/088307380201700406 12088082

[56] Klein-Tasman BP Mervis CB Lord C Phillip K. Socio-communicative deficits in young children with Williams syndrome: performance on the autism diagnostic observation schedule. Child Neuropsychol (2007) 13:444–67. 10.1080/09297040601033680 17805996

[57] Hanley M Riby DM Caswell S Rooney S Back E Looking and thinking: how individuals with Williams syndrome make judgments about mental states. Res Dev Disabil (2013) 34(12):4466–76. 10.1016/j.ridd.2013.09.026 24139712

[58] Porter MA Coltheart M. Cognitive heterogeneity in Williams syndrome. Dev Neuropsychol (2005) 27:275–306. 10.1207/s15326942dn2702_5 15753050

[59] Campbell L McCabe K Leadbeater K Schall U Loughland C Rich D. Visual scanning of faces in 22q11.2 deletion syndrome: attention to the mouth or the eyes? Psychiatry Res (2010) 177(1–2):211–5. 10.1016/j.psychres.2009.06.007 20381171

[60] Campbell LE Stevens AF McCabe K Cruickshank L Morris RG Murphy DGM Is theory of mind related to social dysfunction and emotional problems in 22q11.2 deletion syndrome (velo-cardio-facial syndrome)? J Neurodev Disord (2011) 3(2):152–61. 10.1007/s11689-011-9082-7 21544568 PMC3188292

[61] Campbell LE McCabe KL Melville JL Strutt PA Schall U. Social cognition dysfunction in adolescents with 22q11.2 deletion syndrome (velo-cardio-facial syndrome): relationship with executive functioning and social competence/functioning. J Intellect Disabil Res (2015) 59(9):845–59. 10.1111/jir.12183 25726953

[62] Franchini M Schaer M Glaser B Kott-Radecka M Debanné M Schneider M Visual processing of emotional dynamic faces in 22q11.2 deletion syndrome. J Intellect Disabil Res (2016) 60(4):308–21. 10.1111/jir.12250 26762203

[63] Glaser B Debban M Ottet MC Vuilleumier P Zesiger P Antonarakis SE Eye gaze during face processing in children and adolescents with 22q11.2 deletion syndrome. J Am Acad Child Adolesc Psychiatry (2010) 49(7):665–74. 10.1016/j.jaac.2010.04.004 20610136

[64] Goldenberg PC Calkins ME Richard J McDonald-McGinn D Zackai E Mitra N Computerized neurocognitive profile in young people with 22q11.2 deletion syndrome compared to youths with schizophrenia and at-risk for psychosis. Am J Med Genet (2012) 159B(1):87–93. 10.1002/ajmg.b.32005 22170773 PMC3272485

[65] Ho JS Radoeva PD Jalbrzikowski M Chow C Hopkins J Tran W-C Deficits in mental state attributions in individuals with 22q11.2 deletion syndrome (Velo-cardio-facial syndrome). Autism Res (2012) 5(6):407–18. 10.1002/aur.1252 22962003 PMC3528795

[66] Leleu A Saucourt G Rigard C Chesnoy G Baudouin JY Rossi M Facial emotion perception by intensity in children and adolescents with 22q11.2 deletion syndrome. Eur Child Adolesc Psychiatry (2016) 25(3):297–310. 10.1007/s00787-015-0741-1 26149605

[67] McCabe K Rich D Loughland CM Schall U Campbell LE. Visual scanpath abnormalities in 22q11.2 deletion syndrome: is this a face specific deficit? Psychiatry Res (2011) 189(2):292–8. 10.1016/j.psychres.2011.06.012 21831452

[68] McCabe KL Melville JL Rich D Strutt PA Cooper G Loughland CM Divergent patterns of social cognition performance in autism and 22q11.2 deletion syndrome (22q11DS). J Autism Dev Disord (2013) 43(8):1926–34. 10.1007/s10803-012-1742-2 23292161

[69] McCabe KL Marlin S Cooper G Morris R Schall U Murphy DG Visual perception and processing in children with 22q11.2 deletion syndrome: associations with social cognition measures of face identity and emotion recognition. J Neurodev Disord (2016) 8:30. 10.1186/s11689-016-9164-7 27536336 PMC4988033

[70] Shashi V Veerapandiyan A Schoch K Kwapil T Keshavan M Ip E Social skills and associated psychopathology in children with chromosome 22q11.2 deletion syndrome: implications for interventions. J Intellect Disabil Res (2012) 56(9):865–78. 10.1111/j.1365-2788.2011.01477.x 21883601

[71] Vangkilde A Jepsen JRM Schmock H Olesen C Arnarsdóttir S Baaré WF Associations between social cognition, skills, and function and subclinical negative and positive symptoms in 22q11.2 deletion syndrome. J Neurodev Disord (2016) 8:42. 10.1186/s11689-016-9175-4 27891188 PMC5112709

[72] Bruno JL Garrett AS Quintin E-M Mazaika PK Reiss AL. Aberrant face and gaze habituation in Fragile X syndrome. Am J Psychiatry (2014) 171(10):1099–106. 10.1176/appi.ajp.2014.13111464 24969119 PMC4182125

[73] Cornish K Kogan C Turk J Manly T James N Mills A The emerging Fragile X premutation phenotype: evidence from the domain of social cognition. Brain Cogn (2005) 57(1):53–60. 10.1016/j.bandc.2004.08.020 15629215

[74] Cornish KM Burack JA Rahman A Munir F Russo N Grant C. Theory of mind deficits in children with Fragile X syndrome. J Intellect Disabil Res (2005) 79(Pt 5):372–8. 10.1111/j.1365-2788.2005.00678.x 15817054

[75] Crawford H Moss J Anderson G Oliver C McCleery JP. Implicit discrimination of basic facial expressions of positive/negative emotion in Fragile X syndrome and autism spectrum disorder. Am J Intellect Dev Disabil (2015) 120(4):328–45. 10.1352/1944-7558-120.4.328 26161470

[76] Garner C Callias M Turk J. Executive function and theory of mind performance of boys with Fragile-X syndrome. J Intellect Disabil Res (1999) 43(Pt6):466–74. 10.1046/j.1365-2788.1999.00207.x 10622362

[77] Grant C Apperly I Oliver C. Is theory of mind understanding impaired in males with Fragile X syndrome? J Abnorm Child Psychol (2007) 35(1):17–28. 10.1007/s10802-006-9077-0 17123170

[78] Hagan CC Hoeft F Mackey A Mobbs D Reiss AL. Aberrant neural function during emotion attribution in female subjects with Fragile X syndrome. J Am Acad Child Adolesc Psychiatry (2008) 47(12):1443–54. 10.1097/CHI.0b013e3181886e92 18981933 PMC4820328

[79] Mazzocco MM Pennington BF Hagerman R. Social cognition skills among females with Fragile X. J Autism Dev Disord (1994) 24(4):473–85. 10.1007/BF02172129 7961331

[80] Shaw TA Porter MA. Emotion recognition and visual-scan paths in Fragile X syndrome. J Autism Dev Disord (2013) 43(5):1119–39. 10.1007/s10803-012-1654-1 23015109

[81] Simon EW Finucane BM. Facial emotion identification in males with Fragile X syndrome. Am J Med Genet (1996) 67(1):77–80. 10.1002/(SICI)1096-8628(19960216)67:1<77::AID-AJMG13>3.0.CO;2-M 8678119

[82] Watson C Hoeft F Garrett AS Hall SS Reiss AL. Aberrant brain activation during gaze processing in boys with Fragile X syndrome. Arch Gen Psychiatry (2008) 65(11):1315–23. 10.1001/archpsyc.65.11.1315 18981343 PMC4445973

[83] Williams TA Porter MA Langdon R. Viewing social scenes: a visual scan-path study comparing Fragile X syndrome and Williams syndrome. J Autism Dev Disord (2013) 43(8):1880–94. 10.1007/s10803-012-1737-z 23224515

[84] Wishart JG Cebula KR Willis DS Pitcairn TK. Understanding of facial expressions of emotion by children with intellectual disabilities of differing aetiology. J Intellect Disabil Res (2007) 51(Pt7):551–63. 10.1111/j.1365-2788.2006.00947.x 17537169

[85] Anaki D Zadikov Mor T Gepstein V Hochberg Z. Face perception in women with Turner syndrome and its underlying factors. Neuropsychologia (2016) 90:274–85. 10.1016/j.neuropsychologia.2016.08.024 27565637

[86] Good CD Lawrence K Thomas NS Price CJ Ashburner J Friston KJ Dosage-sensitive X-linked locus influences the development of amygdala and orbitofrontal cortex, and fear recognition in humans. Brain (2003) 126(Pt 11):2431–46. 10.1093/brain/awg242 12958079

[87] Hong DS Bray S Haas BW Hoeft F Reiss AL. Aberrant neurocognitive processing of fear in young girls with Turner syndrome. Soc Cogn Affect Neurosci (2014) 9(3):255–64. 10.1093/scan/nss133 23171616 PMC3980805

[88] Lawrence K Kuntsi J Coleman M Campbell R Skuse D. Face and emotion recognition deficits in Turner syndrome: a possible role for X-linked genes in amygdala development. Neuropsychology (2003) 17(1):39–49. 10.1037/0894-4105.17.1.39 12597072

[89] Mazzola F Seigal A MacAskill A Corden B Lawrence K Skuse DH. Eye tracking and fear recognition deficits in Turner syndrome. Soc Neurosci (2006) 1(3–4):259–69. 10.1080/17470910600989912 18633792

[90] Roelofs RL Wingbermühle E Freriks K Verhaak CM Kessels RP Egger JI. Alexithymia, emotion perception, and social assertiveness in adult women with Noonan and Turner syndromes. Am J Med Genet A (2015) 167A(4):768–76. 10.1002/ajmg.a.37006 25711203

[91] Ross JL Stefanatos GA Kushner H Zinn A Bondy C Roeltgen D. Persistent cognitive deficits in adult women with Turner syndrome. Neurology (2002) 58(2):218–25. 10.1212/WNL.58.2.218 11805247

[92] Ross JL Stefanatos GA Kushner H Bondy C Nelson L Zinn A The effect of genetic differences and ovarian failure: intact cognitive function in adult women with premature ovarian failure versus turner syndrome. J Clin Endocrinol Metab (2004) 89(4):1817–22. 10.1210/jc.2003-031463 15070950

[93] Godbee K Porter MA. Attribution of negative intention in Williams syndrome. Res Dev Disabil (2013) 34(5):1602–12. 10.1016/j.ridd.2013.01.019 23475010

[94] Järvinen A Ng R Crivelli D Arnold AJ Woo-VonHoogenstyn N Bellugi U Relations between social-perceptual ability in multi- and unisensory contexts, autonomic reactivity, and social functioning in individuals with Williams syndrome. Neuropsychologia (2015) 73:127–40. 10.1016/j.neuropsychologia.2015.04.035 26002754 PMC4468004

[95] Järvinen A Ng R Crivelli D Neumann D Arnold AJ Woo-VonHoogenstyn N Social functioning and autonomic nervous system sensitivity across vocal and musical emotion in Williams syndrome and autism spectrum disorder. Dev Psychobiol (2015) 58(1):17–26. 10.1002/dev.21335 26248474 PMC6462219

[96] Järvinen-Pasley A Pollak SD Yam A Hill KJ Grichanik M Mills D Atypical hemispheric asymmetry in the perception of negative human vocalizations in individuals with Williams syndrome. Neuropsychologia (2010) 48(4):1047–52. 10.1016/j.neuropsychologia.2009.12.002 20005238 PMC2847456

[97] Järvinen-Pasley A Vines BW Hill KJ Yam A Grichanik M Mills D Cross-modal influences of affect across social and non-social domains in individuals with Williams syndrome. Neuropsychologia (2010) 48(2):456–66. 10.1016/j.neuropsychologia.2009.10.003 19822162 PMC4096156

[98] John AE Rowe ML Mervis CB. Referential communication skills of children with Williams Syndrome: understanding when messages are not adequate. Am J Intellect Dev Disabil (2009) 114(2):85–99. 10.1352/2009.114.85-99 19391675 PMC2674623

[99] Lacroix A Guidetti M Rogé B Reilly J. Recognition of emotional and nonemotional facial expressions: a comparison between Williams syndrome and autism. Res Dev Disabil (2009) 30(5):976–85. 10.1016/j.ridd.2009.02.002 19286347

[100] Little K Riby DM Janes E Clark F Fleck R Rodgers J. Heterogeneity of social approach behaviour in Williams syndrome: the role of response inhibition. Res Dev Disabil (2013) 34(3):959–67. 10.1016/j.ridd.2012.11.020 23291513

[101] Martínez-Castilla P Burt M Borgatti R Gagliardi C Facial emotion recognition in Williams syndrome and Down syndrome: a matching and developmental study. Child Neuropsychol (2014) 21(5):668–92. 10.1080/09297049.2014.945408 25103548

[102] Mobbs D Garrett AS Menon V Rose FE Bellugi U Reiss AL. Anomalous brain activation during face and gaze processing in Williams syndrome. Neurology (2004) 62(11):2070–6. 10.1212/01.WNL.0000129536.95274.DC 15184616

[103] Plesa-Skwerer D Faja S Schofield C Verbalis A Tager-Flusberg H. Perceiving facial and vocal expressions of emotion in individuals with Williams syndrome. Am J Ment Retard (2006) 111(1):15–26. 10.1352/0895-8017(2006)111[15:PFAVEO]2.0.CO;2 16332153

[104] Porter M Coltheart M Langdon R. The neuropsychological basis of hypersociability in Williams and Down syndrome. Neuropsychologia (2007) 45(12):2839–49. 10.1016/j.neuropsychologia.2007.05.006 17597166

[105] Porter MA Coltheart M Langdon R. Theory of mind in Williams syndrome assessed using a nonverbal task. J Autism Dev Disord (2008) 38(5):806–14. 10.1007/s10803-007-0447-4 17874179

[106] Porter MA Shaw TA Marsh PJ. An unusual attraction to the eyes in Williams-Beuren syndrome: a manipulation of facial affect while measuring face scanpaths. Cogn Neuropsychiatry (2010) 15(6):505–30. 10.1080/13546801003644486 20432078

[107] Riby DM Doherty-Sneddon G Bruce V. Exploring face perception in disorders of development: evidence from Williams syndrome and autism. J Neuropsychol (2008) 2(Pt1):47–64. 10.1348/174866407X255690 19334304

[108] Riby DM Hancock PJ. Do faces capture the attention of individuals with Williams syndrome or autism? Evidence from tracking eye movements. J Autism Dev Disord (2009) 39(3):421–31. 10.1007/s10803-008-0641-z 18787936

[109] Riby DM Hancock PJ Jones N Hanley M. Spontaneous and cued gaze-following in autism and Williams syndrome. J Neurodev Disord (2013) 5(1):13. 10.1186/1866-1955-5-13 23663405 PMC3766200

[110] Santos A Rosset D Deruelle C Human versus non-human face processing: evidence from Williams syndrome. J Autism Dev Disord (2009) 39(11):1552–9. 10.1007/s10803-009-0789-1 19557509

[111] Santos A Deruelle C verbal peaks and visual valleys in theory of mind ability in Williams syndrome. J Autism Dev Disord (2009) 39(4):651–9. 10.1007/s10803-008-0669-0 19039658

[112] Sparaci L Stefanini S Marotta L Vicari S Rizzolatti G. Understanding motor acts and motor intentions in Williams syndrome. Neuropsychologia (2012) 50(7):1639–49. 10.1016/j.neuropsychologia.2012.03.019 22465861

[113] Sparaci L Stefanini S D’Elia L Vicari S Rizzolatti G. What and why understanding in autism spectrum disorders and Williams syndrome: similarities and differences. Autism Res (2014) 7(4):421–32. 10.1002/aur.1370 24604708

[114] Skwerer DP Borum L Verbalis A Schofield C Crawford N Ciciolla L Autonomic responses to dynamic displays of facial expressions in adolescents and adults with Williams syndrome. Soc Cogn Affect Neurosci (2009) 4(1):93–100. 10.1093/scan/nsn041 19047076 PMC2656881

[115] Tager-Flusberg H Sullivan K. A componential view of theory of mind: evidence from Williams syndrome. Cognition (2000) 76(Pt1):59–90. 10.1016/S0010-0277(00)00069-X 10822043

[116] Van Herwegen J Dimitriou D Rundblad G. Performance on verbal and low-verbal false belief tasks: evidence from children with Williams syndrome. J Commun Disord (2013) 46(5–6):440–8. 10.1016/j.jcomdis.2013.10.002 24239484

[117] Widen SC Russell JA. A closer look at preschoolers’ freely produced labels for facial expressions. Dev Psychol (2003) 39(1):114–28. 10.1037/0012-1649.39.1.114 12518813

[118] Djukic A Rose SA Jankowski JJ Feldman JF. Rett syndrome: recognition of facial expression and its relation to scanning patterns. Pediatr Neurol (2014) 51(5):650–6. 10.1016/j.pediatrneurol.2014.07.022 25217338

[119] Williams TA Porter MA Langdon R. Social approach and emotion recognition in Fragile X syndrome. Am J Intellect Dev Disabil (2014) 119(2):133–50. 10.1352/1944-7558-119.2.133 24679350

[120] Van Rijn S Stockmann L van Buggenhout G van Ravenswaaij-Arts C Swaab H. Social cognition and underlying cognitive mechanisms in children with an extra X chromosome: a comparison with autism spectrum disorder. Genes Brain Behav (2014) 13(5):459–67. 10.1111/gbb.12134 24655419

[121] Hernandez N Metzger A Magné R Bonnet-Brilhault F Roux S Barthélémy C Exploration of core features of a human face by healthy and autistic adults analyzed by visual scanning. Neuropsychologia (2009) 47(4):1004–12. 10.1016/j.neuropsychologia.2008.10.023 19027761

[122] Schurgin MW Nelson J Iida S Ohira H Chiao JY Franconeri SL. Eye movements during emotion recognition in faces. J Vis (2014) 14(13):14. 10.1167/14.13.14 25406159

[123] Lo ST Siemensma E Collin P Hokken-Koelega A. Impaired theory of mind and symptoms of autism spectrum disorder in children with Prader-Willi syndrome. Res Dev Disabil (2013) 34(9):2764–73. 10.1016/j.ridd.2013.05.024 23792373

[124] Lewis P Abbeduto L Murphy M Richmond E Giles N Bruno L Cognitive, language and social-cognitive skills of individuals with Fragile X syndrome with and without autism. J Intellect Disabil Res (2006) 50(Pt 7):532–45. 10.1111/j.1365-2788.2006.00803.x 16774638

[125] Sullivan K Tager-Flusberg H Second-order belief attribution in Williams syndrome: intact or impaired? Am J Ment Retard (1999) 104:523–32. 10.1352/0895-8017(1999)104<0523:SBAIWS>2.0.CO;2 10587733 PMC1201457

[126] Steerneman P Meesters C ToM Test-R Handleiding. Antwerpen-Apeldoorn: GarantUitgevers (2009).

[127] Iacoboni M Molnar-Szakacs I Gallese V Buccino G Mazziotta JC Rizzolatti G Grasping the intentions of others with one’s own mirror neuron system. PLoS Biol (2005) 3(3):e79 10.1371/journal.pbio.0030079 15736981 PMC1044835

[128] Riby DM Hanley M Kirk H Clark F Little K Fleck R The interplay between anxiety and social functioning in Williams syndrome. J Autism Dev Disord (2014) 44:1220–9. 10.1007/s10803-013-1984-7 24197115

[129] Hali H Grice SJ Bolton R Johnson MH. Face and gaze processing in Prader-Willi syndrome. J Neuropsychol (2008) 2(Pt 1):65–77. 10.1348/174866407X243305 19334305

[130] Thakkar KN Park S. Empathy, schizotypy, and visuospatial transformations. Cogn Neuropsychiatry (2010) 15(5):477–500. 10.1080/13546801003711350 20437282 PMC2933940

[131] Spreng RN Examining the role of memory in social cognition. Front Psychol (2013) 4:437 10.3389/fpsyg.2013.00437 23874320 PMC3709095

[132] Carlson SM Moses LJ Individual differences in inhibitory control and children’s theory of mind. Child Dev (2001) 72(4):1032–53. 10.1111/1467-8624.00333 11480933

[133] Hobson RP Autism and the Development of Mind. London: Psychology Press (1995). 246 p.

[134] Demily C Rigard C Peyroux E Chesnoy-Servanin G Morel A Franck N «Cognitus & Moi»: a computer-based cognitive remediation program for children with intellectual disability. Front Psychiatry (2016) 7:10 10.3389/FPSYT.2016.00010 26869942 PMC4737901

[135] Glaser B Lothe A Chabloz M Dukes D Pasca C Redoute J Candidate socioemotional remediation program for individuals with intellectual disability. Am J Intellect Dev Disabil (2012) 117(5):368–83. 10.1352/1944-7558-117.5.368 22998485

[136] Ambadar Z Schooler JW Cohn JF. Deciphering the enigmatic face: the importance of facial dynamics in interpreting subtle facial expressions. Psychol Sci (2005) 16(5):403–10. 10.1111/j.0956-7976.2005.01548.x 15869701

[137] Furl N Hadj-Bouziane F Liu N Averbeck BB Ungerleider LG. Dynamic and static facial expressions decoded from motion-sensitive areas in the macaque monkey. J Neurosci (2012) 32(45):15952–62. 10.1523/JNEUROSCI.1992-12.2012 23136433 PMC3539420

[138] Gepner B Deruelle C Grynfeltt S Motion and emotion: a novel approach to the study of face processing by autistic children. J Autism Dev Disord (2001) 31:37–45. 10.1023/A:1005609629218 11439752

[139] Klein-Tasman BP Phillips KD Lord CE Mervis CB Gallo F. Overlap with the autism spectrum in young children with Williams syndrome. J Dev Behav Pediatr (2009) 30(4):289–99. 10.1097/DBP.0b013e3181ad1f9a 19668090 PMC2763277

[140] Shashi V Harrell W Eack S Sanders C McConkie-Rosell A Keshavan MS Social cognitive training in adolescents with chromosome 22q11.2 deletion syndrome: feasibility and preliminary effects of the intervention. J Intellect Disabil Res (2015) 59(10):902–13. 10.1111/jir.12192 25871427 PMC5824427

[141] Fabio RA Castelli I Marchetti A Antonietti A. Training communication abilities in Rett syndrome through reading and writing. Front Psychol (2013) 4:911. 10.3389/fpsyg.2013.00911 24367345 PMC3854542

[142] Antonietti A Castelli I Fabio RA Marchetti A Understanding emotions and mental states from faces and pictures in Rett syndrome. In: Balconi M, editor. Emotional Face Comprehension Neuropsychological Perspectives. New York: Nova Science Publishers (2008). p. 205–32.

[143] Djukic A Valicenti McDermott M Mavrommatis K Martins CL. Rett syndrome: basic features of visual processing-a pilot study of eye-tracking. Pediatr Neurol (2012) 47(1):25–9. 10.1016/j.pediatrneurol.2012.04.009 22704012

[144] Lariviere J Eye Gaze Technology For Girls With Rette Syndrome: From Trials to Conversations. (2014). Available from: http://www.dbmhresource.org/uploads/2/2/5/7/225717

[145] Lariviere JA Eye tracking: eye-gaze technology. 2nd ed In: Söderback I, editor. International Handbook of Occupational Therapy Interventions. Switzerland: Springer International Publishing (2015). p. 339–62.

[146] Townend GS Marschik PB Smeets E van de Berg R van den Berg M Curfs LMG. Eye gaze technology as a form of augmentative and alternative communication for individuals with Rett syndrome: experiences of families in the Netherlands. J Dev Phys Disabil (2016) 28:101–12. 10.1007/s10882-015-9455-z 27069348 PMC4785214

[147] Matsumoto D Keltner D Shiota MN O’Sullivan M Frank M Facial expressions of emotion. 3rd ed In: Lewis M Haviland-Jones JM Barrett LF, editors. Handbook of Emotions. New York: Guilford (2008). p. 211–34.

[148] Tooby J Cosmides L The psychological foundations of culture. In: Barkow J Cosmides L Tooby J, editors. The Adapted Mind: Evolutionary Psychology and the Generation of Culture. New York: Oxford University Press (1992). p. 19–136.

[149] Dezecache G Mercier H Scott-Phillips TC An evolutionary approach to emotional communication. J Pragmatics (2013) 59:221–33. 10.1016/j.pragma.2013.06.007

[150] Marsh AA Ambady N Kleck RE. The effects of fear and anger facial expressions on approach- and avoidance-related behaviors. Emotion (2005) 5(1):119–24. 10.1037/1528-3542.5.1.119 15755225

[151] Winkielman P Berridge KC Wilbarger JL. Unconscious affective reactions to masked happy versus angry faces influence consumption behavior and judgments of value. Pers Soc Psychol Bull (2005) 31(1):121–35. 10.1177/0146167204271309 15574667

